# The deubiquitinase Leon/USP5 interacts with Atg1/ULK1 and antagonizes autophagy

**DOI:** 10.1038/s41419-023-06062-x

**Published:** 2023-08-22

**Authors:** Yueh-Ling Pai, Yuchieh Jay Lin, Wen-Hsin Peng, Li-Ting Huang, He-Yen Chou, Chien-Hsiang Wang, Cheng-Ting Chien, Guang-Chao Chen

**Affiliations:** 1 Institute of Biological Chemistry, Academia Sinica, Taipei, 115 Taiwan; 2 Institute of Biochemical Sciences, College of Life Science, National Taiwan University, Taipei, 106 Taiwan; 3 Chemical Biology and Molecular Biophysics, Taiwan International Graduate Program, Academia Sinica, Taipei, 115 Taiwan; 4 Institute of Molecular Biology, Academia Sinica, Taipei, 115 Taiwan

**Keywords:** Macroautophagy, Deubiquitylating enzymes

## Abstract

Accumulating evidence has shown that the quality of proteins must be tightly monitored and controlled to maintain cellular proteostasis. Misfolded proteins and protein aggregates are targeted for degradation through the ubiquitin proteasome (UPS) and autophagy-lysosome systems. The ubiquitination and deubiquitinating enzymes (DUBs) have been reported to play pivotal roles in the regulation of the UPS system. However, the function of DUBs in the regulation of autophagy remain to be elucidated. In this study, we found that knockdown of Leon/USP5 caused a marked increase in the formation of autophagosomes and autophagic flux under well-fed conditions. Genetic analysis revealed that overexpression of Leon suppressed Atg1-induced cell death in *Drosophila*. Immunoblotting assays further showed a strong interaction between Leon/USP5 and the autophagy initiating kinase Atg1/ULK1. Depletion of Leon/USP5 led to increased levels of Atg1/ULK1. Our findings indicate that Leon/USP5 is an autophagic DUB that interacts with Atg1/ULK1, negatively regulating the autophagic process.

## Introduction

Cellular homeostasis, maintained by a well-balanced control of protein synthesis and degradation, is essential for normal cell growth and development. The ubiquitin-proteasome system (UPS) and autophagy are the two main intracellular degradative systems in eukaryotes [1]. UPS mainly degrades specific short-lived proteins, whereas autophagy is responsible for the bulk degradation of long-lived proteins and damaged organelles [2, 3]. Autophagy plays pivotal roles in maintaining cellular homeostasis in response to environmental stresses such as nutrient deprivation, ROS and pathogen invasion [4]. Dysregulation of autophagy pathway has been associated with a variety of human diseases, including neurodegeneration, immunity, cardiovascular diseases, and cancer [5, 6].

The protein posttranslational modifications (PTMs) have emerged as potent regulators of autophagy. Ubiquitination is one of the most versatile form of PTM to control protein stability, activity, localization and interaction with other cellular components [7]. Aside from the two ubiquitin-like conjugation systems (Atg12-Atg5 and Atg8/LC3), accumulating evidence has shown that ubiquitination plays an important role in modulating the functions of Atg proteins [8, 9]. Moreover, ubiquitination is also essential for substrate targeting during selective autophagy. Ubiquitinated protein aggregates, pathogens [10], and damaged organelles such as mitochondria [11] and ER [12] are recognized by specific autophagy receptors for lysosomal degradation [13].

Deubiquitinases (DUBs) are a group of enzymes responsible for the proteolytic removal of ubiquitin molecule from ubiquitin chains and ubiquitinated proteins [14, 15]. More than 90 DUBs have been identified in human genome. Based on their sequence similarities and likely mechanisms of action, DUBs are classified into five distinct subfamilies: ubiquitin specific proteases (USPs), ubiquitin C-terminal hydrolases (UCHs), ovarian tumor proteases (OTUs), Machado-Joseph disease proteases (MJDs) and JAB1/MPN/Mov34 metalloisopeptidases (JAMMs) [14]. While DUBs have been implicated in regulating diverse cellular pathways and diseases [15, 16], the role of DUBs in autophagy is just starting to emerge. Shi and Kehrl showed that the OTU-type deubiquitinase A20 antagonizes TRAF6-mediated ubiquitination of Beclin-1 upon TLR4 activation, and thus impairs autophagy [17]. Additionally, several USP family DUBs, including USP11, USP14, and USP19, were found to maintain the stability of Beclin-1 during autophagy [18–20]. It was also reported that USP20 promotes autophagy initiation by stabilizing ULK1 [21]. Moreover, recent studies have implicated the involvement of DUBs in selective autophagy. It has been shown that USP36 depletion leads to the accumulation of ubiquitinated proteins and induces selective autophagy via a p62-dependent pathway [22]. Bingol et al. showed that USP30, a deubiquitinase localized in mitochondria, counteracts Parkin-mediated mitophagy [23]. Conversely, USP8 specifically cleaves Lys6-linked ubiquitin conjugates from Parkin and activates Parkin-dependent mitophagy [24].

In this study, we performed a genetic screen (RNAi knockdown) to identify *Drosophila* DUBs involved in the regulation of autophagy. We found that knockdown of *Drosophila Leon/USP5* leads to increased autophagosome formation and autophagic flux. Leon depletion resulted in an accumulation of Ref(2)P/p62 and polyubiquitinated protein aggregates. We further discovered that Leon genetically and biochemically interacted with Atg1 to regulate autophagy, and loss of *leon* caused increased Atg1 levels. In mammals, depletion of USP5 also caused activation of autophagy. USP5 interacts with ULK1 and regulates its expression. These findings indicate an evolutionary conversed role of Leon/USP5-Atg1/ULK1 axis in the regulation of autophagy.

## Results

### Leon/USP5 acts as a negative regulator of autophagy

**Fig. 1:**
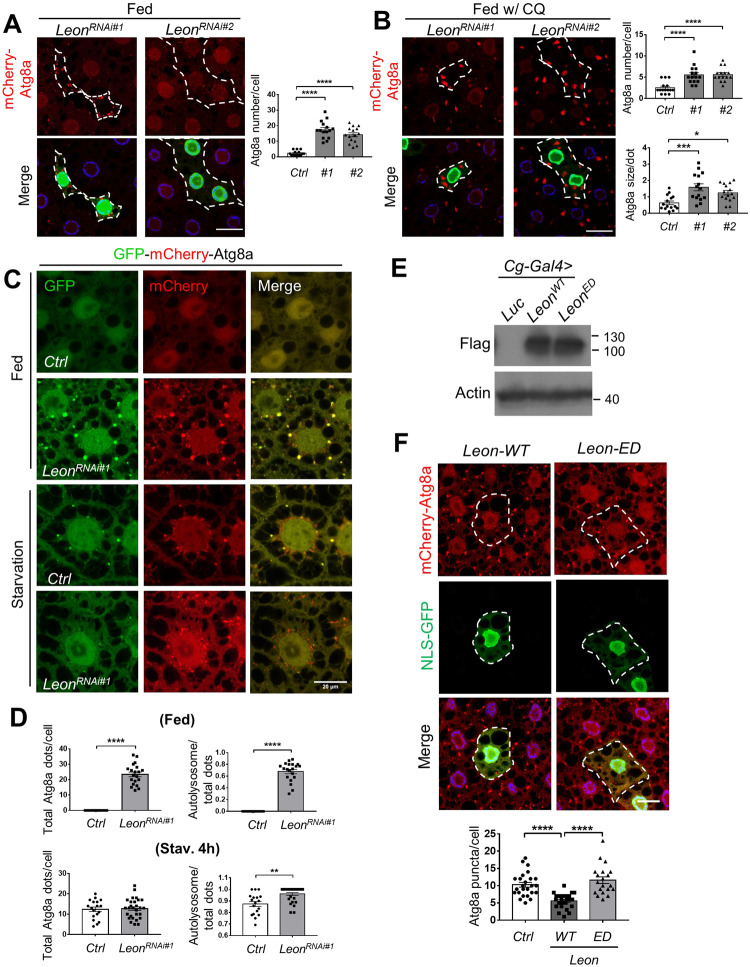
Leon depletion enhances autophagosome formation and autophagic flux. **A**, **B** Clonal knockdown of *Leon* (GFP positive) in the larval fat bodies using the flp-out system caused an increase in mCherry-Atg8a puncta, compared with controls (GFP negative). *Drosophila* second instar larvae were reared on media without (**A**) or with chloroquine (**B**). Quantitative data showed the number of Atg8a puncta in control and *Leon* knockdown cells. Data shown as mean ± SEM; *n* ≥ 10 larvae, ≥ 15 cells per genotype. **C** Depletion of Leon promotes autophagy flux. Confocal microscopy analysis of control and *Leon* knockdown early third instar larval fat body expressing *GFP-mCherry-Atg8a* with *Cg-Gal4* under fed and starvation conditions. **D** Quantification of number of total Atg8a puncta and ratio of autolysosomes (GFP^-^ mCherry^+^) to autophagosomes (GFP^+^ mCherry^+^) per cell. *n* ≥ 15 larvae, ≥ 20 cells per genotype. **E** Immunoblot analysis of Flag-tagged Leon-WT (wild-type) and Leon-DE (enzyme dead) expression driven by *Cg-Gal4* driver. **F** Clonal expression of *Leon-WT* but not *Leon-ED* (GFP positive) in the *Drosophila* larval fat bodies caused a decrease in starvation-induced mCherry-Atg8a puncta formation, compared with controls (GFP negative). Quantification of Atg8a puncta number per cell. Scale bar, 20 μm. N ≥ 20 cells. Data shown as mean ± SEM of three independent experiments, *P < 0.05; **P < 0.01; ***P < 0.001; ****P < 0.0001.

To identify novel DUBs involved in the regulation of autophagy, we conducted a targeted RNAi screen in *Drosophila* larval fat body. The *Drosophila* fat body is the major nutrient storage organ similar to the mammalian liver and adipose tissues, and autophagy is dramatically activated upon nutrient starvation [25]. Using the Flp-FRT system [26], we clonal knock down of 33 *Drosophila* DUBs in larval fat body cells expressing the autophagy marker mCherry-Atg8a (Table S1). In control cells, the mCherry-Atg8a showed a diffused cytoplasmic distribution under nutrient-rich conditions and became localized to punctate structure in response to starvation (Fig. 1A). Ablation of four DUBs, including *CG12082/Leon* (*USP5*), *CG5798/Ubpy* (*USP8*), *CG5505/Scny* (*USP36*), and *CG4165* (*USP45*), caused increased number of Atg8a puncta under fed conditions, whereas *CG7288* (*USP39*) depletion resulted in decreased Atg8a puncta formation under both fed and starvation conditions. Because previous studies have reported the function of Ubpy, Usp12 and Scny in autophagy [22, 27, 28], here we focus on the role of Leon/USP5 in autophagy. Clonal knockdown of *Leon* with two different RNAi lines result in the accumulation of mCherry-Atg8a puncta under nutrient-rich conditions (Fig. 1A, S1A). Inhibition of autophagy machinery by coexpression of *Atg7*^*RNAi*^ suppressed the *Leon*^*RNAi*^ induced Atg8a puncta formation (Fig. S1B), suggesting a role of Leon in the regulation of autophagy. We also generated the *leon* mitotic mutant clones in the larval fat bodies and stained them with the autolysosomal marker Lysotracker Red to assess lysosomal activity. The *leon* mutant clones exhibit a dramatic increase of punctate Lysotracker staining (Fig. S1C), indicating an accumulation of acidic autolysosomes. The increased number of mCherry-Atg8a puncta in *Leon*-knockdown cells could be due to either the induction of autophagy or impaired autophagy flux. To determine the involvement of Leon in autophagy, we first fed the larvae with the lysosome inhibitor chloroquine (CQ) under fed conditions. As shown in Fig. 1B, CQ treatment significantly increased the number of mCherry-Atg8a puncta in Leon depleted cells, compared to controls. We further checked the effect of Leon depletion on starvation-induced autophagy and autophagic flux using the tandem fluorescent-tagged GFP-mCherry-Atg8a reporter. The tandem reporter emits both red and green fluorescence in neutral autophagosomes, whereas the GFP fluorescence is quickly quenched, leaving only red fluorescence, in acidic autolysosomes. We found a markedly increased number of total Atg8a puncta and enhanced autophagic flux in Leon depleted cells, compared to controls (Fig. 1C, D), suggesting that ablation of Leon leads to the activation of autophagy. Next, we checked whether the overexpression of Leon affects autophagy. As shown in Fig. 1E, F, we found that clonal expression of *Leon-WT* but not the catalytically inactive mutant *Leon-ED* caused a significant decreased in Atg8a puncta formation under starvation conditions. Collectively, these results indicate that Leon plays a negative role in the regulation of autophagosome formation and autophagic flux.

### Loss of *Leon* leads to accumulation of Ref(2)P and polyubiquitinated protein aggregates

**Fig. 2:**
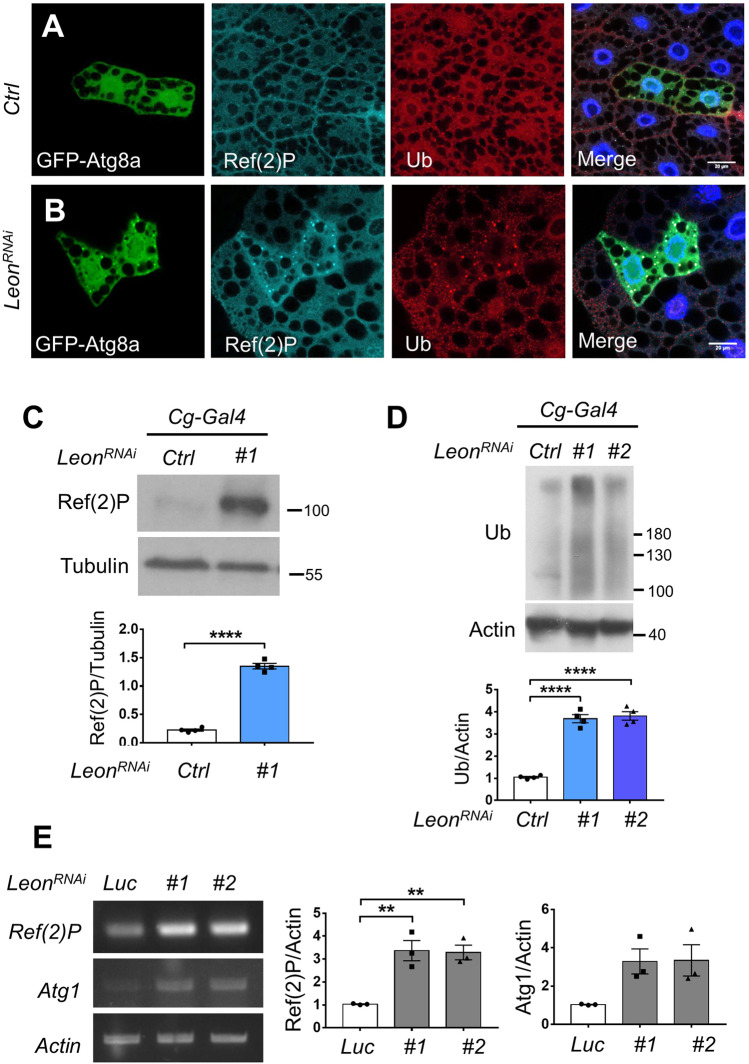
Leon depletion leads to the accumulation of Ref(2)P and polyubiquitinated protein aggregates. **A** Clonal expression *GFP-Atg8a* in *Drosophila* larval fat body under fed conditions. **B** Clonal expression of *Leon*^*RNAi*^ resulted in the formation of Atg8a puncta and an increased number of Ref(2)P puncta and ubiquitin-positive aggregates. **C**, **D** Western blot analyses of Ref(2)P (**C**) ubiquitinated protein (**D**) expression levels in the *Drosophila* larval fat bodies expressing *GFP* (Ctrl) or *Leon*^*RNAi*^ under the control of *Cg-Gal4*. Blots are representative of three independent experiments. Data are presented as means ± SEMs. ****P < 0.0001. **E** RT-PCR analysis revealed that Leon knockdown caused increased levels of *Ref(2)P* and *Atg1* mRNA in larval fat body. Quantification of *Ref(2)P* and *Atg1* expression normalized to *Actin*. Data as shown by mean ± SEM from three independent experiments, **p < 0.01.

**Fig. 3:**
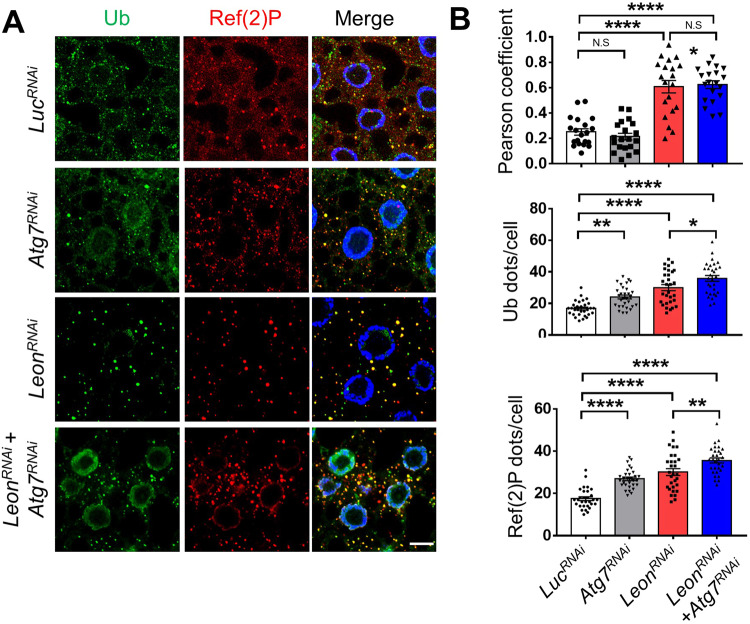
Depletion of Atg7 enhances Ref(2)P puncta formation and ployubiquitinated protein aggregates in Leon-depleted cells. **A** Confocal microscopy analysis of larval fat body expressing *Luc*^*RNAi*^, *Leon*^*RNAi*^, *Atg7*^*RNAi*^ or co-expressing *Leon*^*RNAi*^ and *Atg7*^*RNAi*^ with *Cg-Gal4* driver and stained with Ub and Ref(2)P antibodies. **B** Quantification of the number and colocalization of Ref(2)P puncta and polyubiquitin aggregates in (**A**). The Pearson’s correlation coefficient was analyzed by imageJ. Scale bar, 10μm. Data as shown by mean ± SEM in *N* = 30 cells from three independent experiments, **P < 0.01; **** *P* < 0.0001; NS not significant.

Our previous study has shown that Leon plays a critical role in maintaining ubiquitin homeostasis during *Drosophila* development [29]. Loss of *Leon* leads to proteasomal degradation defects and accumulation of polyubiquitinated proteins. Several recent studies have shown that proteasome dysfunction results in increased expression of *p62*/*SQSTM1* and *ATG* genes, enhanced autophagic flux, and the formation of aggregates positive for p62*/*SQSTM1 and ubiquitin [30–32]. Consistently, clonal depletion of *Leon* in the larval fat body resulted in the accumulation of polyubiquitinated protein aggregates (Fig. 2A, B). The expression levels of Ref(2)P, the *Drosophila* homolog of mammalian p62/SQSTM1, were also dramatically increased in Leon depleted cells, compared to controls (Fig. 2A–C, E). Immunofluorescence analysis further revealed a high degree of colocalization between Atg8a, Ref(2)P puncta and polyubiquitinated protein aggregates in Leon depleted cells (Figs. 2B, 3A). Similarly, analysis of the mitotic *leon* null clones showed dramatically increased polyubiquitinated protein aggregates and Ref(2)P puncta (Fig. S2). In addition to being an autophagy substrate, p62/SQSTM1 also acts as a cargo receptors for degradation of ubiquitinated proteins [33]. The increased Ref(2)P may contribute to the sequestration of polyubiquitinated protein aggregates for selective autophagic degradation in Leon-depleted cells. We further examined whether inhibition of autophagy may lead to more aggregates of Ref(2)P and ubiquitinated proteins caused by Leon depletion. As shown in Fig. 3A, B, there are significantly increased number of Ref(2)P and ubiquitinated protein aggregates in both *Leon* and *Atg7* knockdown cells compared to *Leon* knockdown cells.

### Leon interacts with the autophagy initiator Atg1

**Fig. 4:**
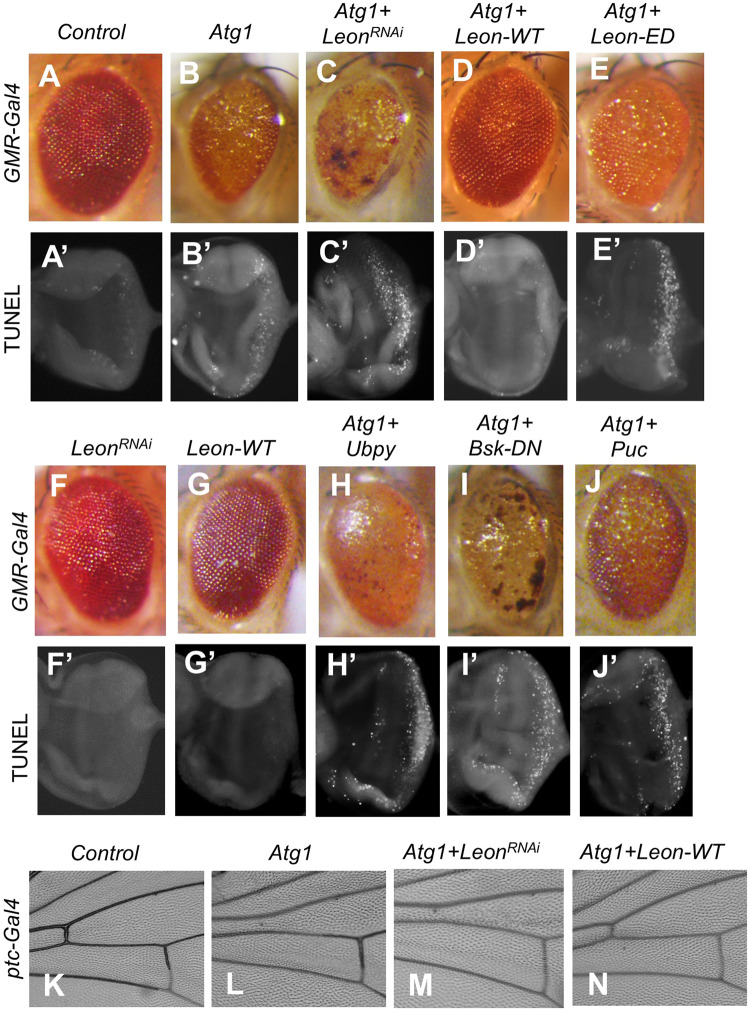
Overexpression of Leon suppresses Atg1-induced eye and wing defects. **A**–**J** The Atg1-induced rough eye phenotypes (**A**–**J**) and cell death (**A**’–**J**’) were rescued by coexpression of *Leon-WT* (**D**), but not the catalytically inactive *Leon-ED* (**E**), *Leon*^*RNAi*^ (**C**), *Ubpy* (**H**), dominant negative Bsk (*Bsk*^DN^) (**J**), or *Puc* (**J**). Third-instar larval eye imaginal discs from *GMR-Gal4* controls or flies expressing indicated transgenes were stained with TUNEL to detect relative levels of cell death (**A**′–**J**′). **K**–**N** Compare with the *ptc-Gal4* control (**K**), expression *Atg1* (**L**) by *ptc‐Gal4* resulted in anterior cross‐vein missing phenotypes. The Atg1-induced wing vein defects were rescued by coexpression of *Leon-WT* (**N**), but not *Leon*^*RNAi*^ (**M**).

Atg1 Ser/Thr kinase acts as a key regulator in the initiation of autophagy. We and others have previously shown that overexpression of *Drosophila* Atg1 induces autophagy and cell death [34, 35]. Interestingly, we found that Atg1-induced rough eye phenotypes could be rescued by coexpression of wild-type (WT) but not the enzyme-dead (ED) form of Leon in the developing eye, whereas knockdown of *Leon* enhanced Atg1-induced eye defects (Fig. 4A–E), suggesting that the Leon-ED mutant may not have a dominant negative effect. Similarly, TUNEL assays in the third-instar larval eye imaginal discs revealed that coexpression of *Leon-WT* but not *Leon-ED* or *Leon*^*RNAi*^ suppressed Atg1-induced cell death (Fig. 4A’–E’). Moreover, we found that overexpression of *Ubpy/USP8*, an autophagy-related DUB, could not suppress the Atg1-induced eye defects (Fig. 4H, H’), suggesting that the interaction between Leon and Atg1 is DUB specific. It has been reported that Leon negatively regulates JNK signaling and apoptosis during *Drosophila* eye development [36]. We next checked whether Leon suppressed Atg1-induced rough eye phenotypes by inhibiting the JNK signaling pathway. Co-expression of dominant negative JNK (*Bsk-DN*) or JNK phosphatase *Puc* could not rescue the Atg1-induced cell death and eye defects (Fig. 4 I, J, I’, J’), suggesting that Leon interacts with Atg1 via a JNK signaling independent manner.

To determine whether the interaction between Leon and Atg1 is tissue specific, we next checked their interaction in developing wing. Overexpression of *Atg1* by *ptc-Gal4* induced Atg8a puncta formation and caused aberrant actin cytoskeleton organization in larval wing imaginal discs and the loss of anterior cross vein in adult wings (Fig. 4K, L and S3A, B, A’, B’). Coexpression of *Leon-WT* dramatically reduced the number of Atg8a puncta and suppressed actin cytoskeleton defects and wing vein loss phenotypes, whereas *Leon* knockdown enhanced the Atg1-induced wing defects (Fig. 4M, N, S3B–D, B’–D’). These results together indicate that *Leon* genetically interacts with *Atg1* to regulate autophagy.

**Fig. 5:**
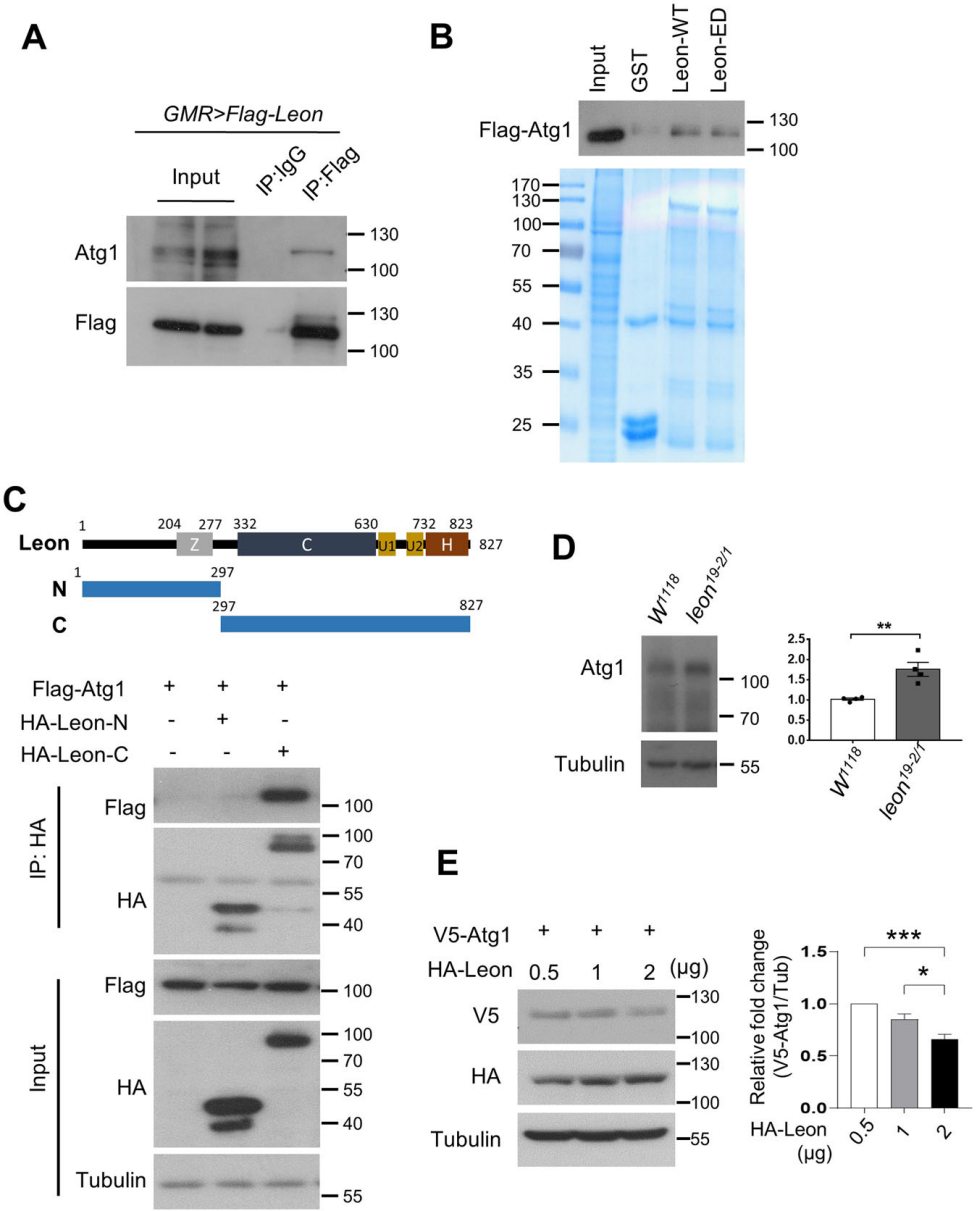
Leon interacts with the autophagy initiator Atg1. **A** Immunoprecipitation analysis of the interaction between Leon and endogenous Atg1 in flies expressing *Flag-Leon* with *GMR-Gal4*. *Drosophila* adult head lysates were immunoprecipitated with anti-IgG or anti-Flag antibody and analyzed by immunoblotting with the indicated antibodies. **B** GST pull*-*down assay to determine Leon-Atg1 interaction. Bacterially expressed GST, GST-Leon-WT and GST-Leon-ED proteins were incubated with cell lysates derived from HEK293T cells expressing Flag-tagged Atg1. The input and pull-down products were analyzed by immunoblotting with the Flag antibody. The relative levels of GST fusion proteins are shown by Coomassie blue staining (bottom). **C** Co-immunoprecipitation assays to map the interaction regions between Atg1 and Leon. HEK293T cells transiently transfected with Flag-Atg1, HA-Leon-N or HA-Leon-C were lysed and subjected to immunoprecipitations and immunoblotting analysis. Domain structures and deletion mutants of Leon are schematically presented. Z, zinc-finger ubiquitin binding domain; C, peptidase domain; U1/U2, ubiquitin-associated domains; H, histidine box. **D** Western blot analysis of Atg1 expression levels in control and *leon*^*19-2/1*^ mutant larvae. **E** Western blot analysis of Atg1 expression levels in HEK293T cells transfected with V5-Atg1 and increasing amounts of HA-Leon. Blots are representative of three independent experiments. Data are presented as means ± SEMs. *P < 0.05; **P < 0.01; ***P < 0.001.

To further elucidate the interaction between Leon and Atg1, we investigated whether Leon physically interacted with Atg1. Immunoblotting of the anti-Flag immunoprecipitates from fly adult head lysates that expressed Flag-tagged Leon revealed co-precipitation between Leon and endogenous Atg1 (Fig. 5A). GST pull-down assays also showed that both GST-Leon-WT and GST-Leon-ED could interact with Atg1 (Fig. 5B), suggesting that the DUB activity of Leon is not required for its association with Atg1. To determine which domain of Leon interacts with Atg1, HEK 293 T cells were transfected with HA-tagged N- or C-terminus Leon together with Flag-tagged Atg1. Co-IP analysis revealed that Leon-C but not Leon-N co-immunoprecipitated with Atg1 (Fig. 5C). We next examined whether Leon co-localizes with Atg1/ULK1 in *Drosophila* larval fat body cells. Immunofluorescence analysis revealed that under fed conditions, Leon is enriched in the nucleus (Fig. S4). Interestingly, under starvation conditions, Leon exhibited cytoplasmic punctate staining that partially co-localized with Atg1/ULK1 (Fig. S4). Besides its role in maintaining cellular ubiquitin homeostasis, mammalian USP5 has been shown to regulate protein stability [37]. We thus checked whether Leon regulated Atg1 levels. As shown in Fig. 5D, we observed increased levels of Atg1 protein in *leon* mutant animals. Whereas ectopic expression of Leon caused a dramatic decrease in the expression of Atg1 in a dose-dependent manner (Fig. 5E). All together, these data indicate that Leon interacts with and regulates Atg1 expression.

### Mammalian USP5 interacts with ULK1 to regulate autophagy

**Fig. 6:**
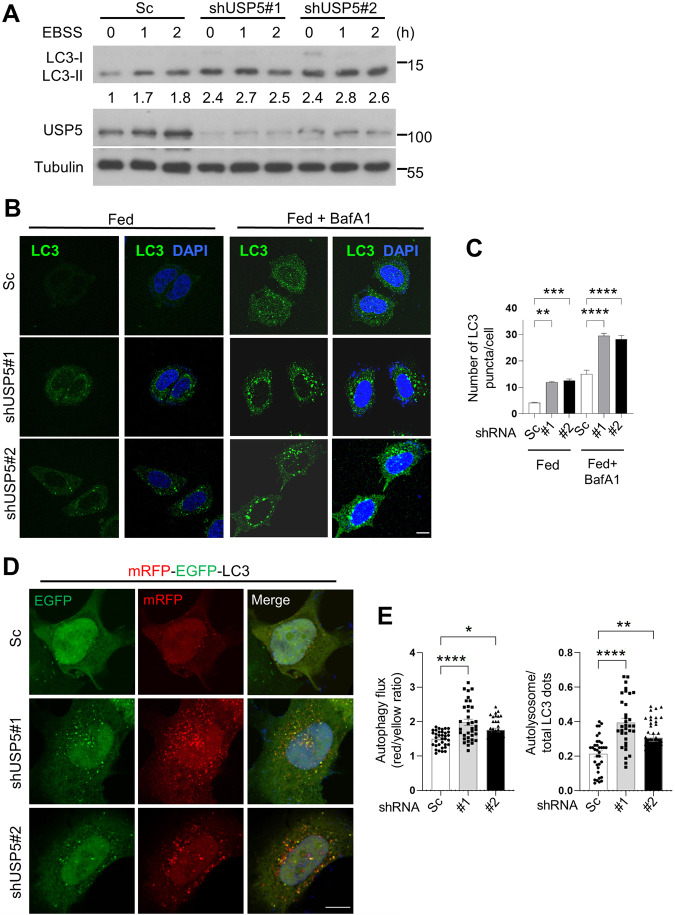
Depletion of USP5 promotes autophagosome formation and autophagic flux. **A** The effects of USP5 depletion on starvation-induced LC3 conversion were assessed by immunoblotting with antibodies as indicated. Control and shUSP5 MCF7 cells were cultured in EBSS for the indicated time points. Numbers below lanes indicate the relative ratio of LC3-II/Tubulin. **B** MCF7 cells stable expressing scramble (Sc) or USP5 shRNA were cultured for 2 h in normal culture medium (Fed) with or without 100 nM bafilomycin A1 (BafA1) and immunostained with LC3 antibody. Nuclei were stained with DAPI. Scale bar: 5 μm. **C** Quantification of the number of LC3 puncta in control (Sc) and USP5 shRNA cells treated as (**A**); data shown as mean ± SEM, *n* = 3, ≥ 35 cells. **D** Immunofluorescence analysis of autophagic flux in mRFP-EGFP-LC3 transfected control or USP5 knockdown cells. **E** Quantification of the ratio of acidic autolysosomes (red) to autophagosomes (yellow) and the ratio of autolysosomes to total LC3 puncta (*n* = 3, ≥30 cells/condition). Scale bar, 10 μm. *P < 0.05; **P < 0.01; ****P < 0.0001.

**Fig. 7:**
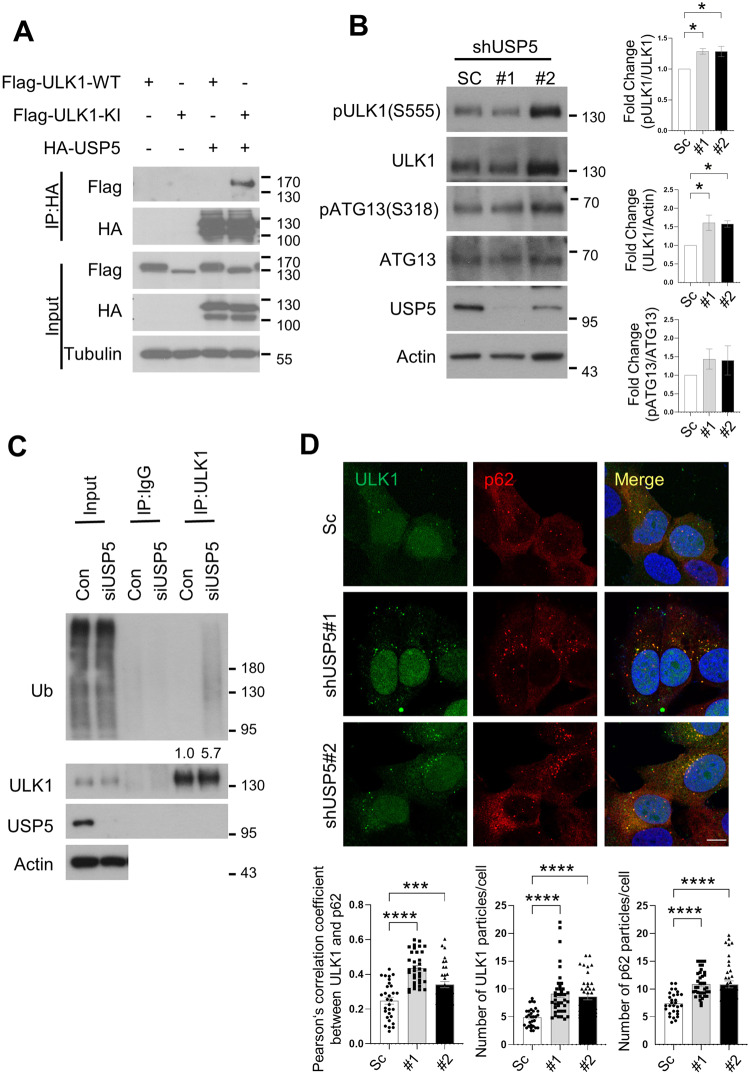
Mammalian USP5 interacts with ULK1 to regulate autophagy. **A** Co-immunoprecipitation analysis of the interaction between USP5 and ULK1. HEK293T transiently transfect with HA-USP5 and Flag-ULK1-WT or Flag-ULK1 catalytically inactive mutant (KI) were subjected to immunoprecipitation with anti-HA antibody. The immunoprecipitates and input were analyzed by immunoblotting with antibodies as indicated. **B** Western blot analysis of ULK1 and pULK1 levels in control (Sc) and USP5 depleted MCF7 cells. The ratios of ULK1 to actin, phospho-ULK1 to ULK1 and phospho-Atg13 to Atg13 were quantified using ImageJ. Blots are representative of three independent experiments. Data are presented as means ± SEM. *P < 0.05. **C** Immunoprecipitation analysis for endogenous ULK1 ubiquitination in control or USP5 knockdown HeLa cells treated with 10 µM MG132 for 6 h. Blots are representative of two biological replicates. **D** Confocal microscopy analysis for the co-localization of ULK1 with p62 in control or USP5 knockdown cells. The bottom panels show the quantification of number and colocalization of ULK1 and p62 puncta in ≥ 30 cells. The Pearson’s correlation coefficient was analyzed by ImageJ. Data are presented as means ± SEM. Scale bar, 10 μm. ****p < 0.0001.

Given that Leon is highly homologous to mammalian USP5, we next investigated whether USP5 also played a role in the regulation of autophagy. As shown in Fig. 6A, LC3 conversion assays revealed an increased levels of LC3-II in USP5-depleted cells under normal and starvation conditions, compared to controls. Similarly, immunofluorescence analysis showed that knockdown of USP5 resulted in dramatically increased LC3 puncta formation with or without the treatment of lysosomal inhibitor bafilomycin A1 (BafA1) (Fig. 6B, C). We further examined the effects of USP5 knockdown on autophagic flux by the tandem mRFP-GFP-LC3 fluorescence analysis. Consistent with our earlier observations, ablation of USP5 significantly increased the autolysosome (red, mRFP^+^ GFP^−^) to autophagosome (yellow, mRFP^+^ GFP^+^) ratio, compared to controls (Fig. 6D, E), suggesting that USP5 plays a negative role in the regulation of autophagic flux and autophagosome formation. Next, we examined whether USP5 interacts with ULK1 and regulates its expression. HEK293T cells transfected with HA-USP5 and Flag-ULK1-WT or Flag-ULK1-KI (catalytically inactive form) were subjected to immunoprecipitations. Immunoblotting of the anti-HA immunoprecipitates from cell lysates showed that USP5 preferentially associated with the ULK1-KI mutant (Fig. 7A). Notably, depletion of USP5 caused a dramatically increased levels of ULK1, pULK1 (Fig. 7B), ubiquitinated ULK1 (Fig. 7C) and ULK1 mRNA expression (Fig. S5B, C), suggesting that USP5 regulates ULK1 at both protein and mRNA levels. We further checked whether USP5 can co-localize with ULK1 in mammalian cells. As shown in Fig. S6, we found that USP5 forms punctate structures that are co-localized with ULK1. Similar to our findings in *Drosophila*, USP5 depletion resulted in increased p62 puncta formation and ubiquitinated protein aggregates and elevated expression of *p62* and *ATG* genes (Fig. S5A–C). Immunofluorescence analysis also revealed a significantly enhanced co-localization of ULK1 and p62 in USP5-depleted cells (Fig. 7D). These results together suggest that USP5 interacts with and regulate ULK1 levels in mammalian cells.

## Discussion

The ubiquitin-proteasome system (UPS) and autophagy are two major cellular protein quality control pathways. Previous studies have shown that impairment of the UPS is often compensated by the upregulation of autophagy [38, 39]. Autophagy eliminates abnormal protein accumulation caused by UPS downregulation to protect cells from undergoing apoptosis. However, the crosstalk between autophagy and UPS is still poorly understood. Previous studies have shown that Leon and its mammalian homolog USP5 specifically recognize and cleave unanchored polyubiquitin chains to maintain cellular ubiquitin homeostasis [29, 40]. Loss of *Leon* leads to accumulation of polyubiquitin, polyubiquitinated proteins as well as impaired proteasomal degradation. In this study, we identified the involvement of Leon/USP5 in the regulation of autophagy. Ablation of Leon/USP5 resulted in the increased number of Atg8a/LC3 puncta and enhanced autophagic flux in *Drosophila* and mammalian cells. Our findings indicate that Leon/USP5 may play a critical role in linking UPS and autophagy pathways.

Leon is essential for animal survival and normal tissue development in *Drosophila*. Loss of Leon function causes increased apoptotic cell death and the activation of JNK pathway in larval imaginal discs [36, 40]. The JNK signaling pathway regulates a range of cellular responses, including apoptosis and autophagy [41]. It was reported that activation of JNK signaling by expression of constitutively activated Hemipterous (*Hep*^*act*^, the *Drosophila* JNKK) stimulates the expression of *Atg* genes and autophagy induction [42]. Autophagy is activated downstream of JNK signaling and oxidative stress in maintaining *Drosophila* intestinal homeostasis [42]. Here we found that Leon genetically interacts with the autophagy initiating kinase Atg1. Ectopic expression of Atg1 results in autophagy activation and autophagic cell death in developing eye and wing, and overexpression of Leon suppressed Atg1-induced eye and wing defects. However, our results also showed that overexpression of a dominant negative form of JNK (*Bsk*^*DN*^) or JNK phosphatase *Puc* could not rescue the Atg1-induced eye defects, suggesting that Leon interacts with Atg1 through a mechanism independent of the activation of JNK signaling pathway.

The Atg1/ULK1 Ser/Thr kinase plays a key role in the induction of autophagy. Our results showed that overexpression of Leon-WT but not the enzyme dead Leon-ED caused a significant decrease in Atg8a puncta formation under starvation conditions. Moreover, co-expression of Leon-WT but not Leon-ED suppressed Atg1-induced cell death, suggesting that the Leon deubiquitinase activity is important for its function in autophagy. Recent studies have shown that the ubiquitination and deubiquitination of ULK1 are critical in the regulation of ULK1 stability and autophagy activity. The TRAF6-mediated K63-linked polyubiquitination of ULK1 promotes activation of autophagy by stabilizing ULK1 [43]. Conversely, the NEDD4L and Cul3-KLHL20 ubiquitin ligases ubiquitylate ULK1 for proteasomal degradation and autophagy termination [44, 45]. In mammals, several DUBs have also been found to regulate the stability of Atg1/ULK1. While the deubiquitinating enzyme USP20 and STAMBP/AMSH promote autophagy initiation by stabilizing ULK1 [21, 46], USP24 was found to negatively regulate autophagy and the stability of ULK1 [47]. Moreover, it was reported that USP1 regulates autophagy by modulating ULK1 ubiquitination and cellular compartmentalization [48]. Here we showed that Leon interacts and regulate Atg1 expression in *Drosophila*. Similarly, we found that mammalian USP5 interacted with ULK1 in the co-IP assays and USP5 depletion caused increased levels of ULK1, pULK1, and *ULK1* mRNA suggesting an evolutionary conserved role of Leon/USP5-Atg1/ULK1 interaction in *Drosophila* and mammals. While USP5 depletion caused increased levels of ubiquitinated ULK1, we have not been able to demonstrate whether Leon/USP5 directly modulates the ubiquitination of Atg1/ULK1. The detailed mechanisms of Leon/USP5-Atg1/ULK1 interaction remain to be elucidated.

Besides its role in development, USP5 has been shown to play crucial roles in diverse cellular physiological and pathological functions. USP5 interacts and promotes the activity of Ca_v_3.2 channel, thereby modulating the inflammatory and neuropathic pain in various mouse models [49, 50]. USP5 also plays an oncogenic role in a variety of cancers, including glioblastoma, lung cancer, hepatocellular carcinoma (HCC), ovarian and pancreatic cancer [37]. Several recent studies have implicated the involvement of USP5 in the regulation of tumor cell growth [51, 52], which may account for the observation of some Leon depleted cells are smaller than control neighboring cells in *Drosophila*. Moreover, in non-small cell lung cancer (NSCLC) cells, USP5 enhances the protein stability of cyclin D1 and PD-L1, and promotes NSCLC cell proliferation and progression [53, 54]. In HCC cells, depletion of USP5 decreases the expression of SLUG and inhibits HCC cell proliferation and invasion [55]. Conversely, recent studies have implicated a tumor suppressive role of ULK1 and autophagy in various cancer types [56, 57]. Our findings indicate an antagonistic role of Leon/USP5 in Atg1/ULK1-mediated autophagy and may provide mechanistic insights into how USP5 promoted tumor growth and progression.

## Methods

### *Drosophila* stocks, genetics and treatment

Flies were raised at 25 °C following standard procedures. The fly strains used in this study were as follows: *GMR-Gal4*, *ptc-Gal4*, *Cg-Gal4* (BL7011), *UASp-mCherry-GFP-Atg8a* (BL37749), *UAS-Atg7*^*RNAi*^ (BL27707), and *UAS-Leon*^*RNAi#1*^ (BL31886) were obtained from the Bloomington Stock Center; *Leon*^*RNAi#2*^ (v17567) was obtained from the Vienna *Drosophila* Resource Center (VDRC); *UAS-Atg1, UAS-Flag-Leon, UAS-Flag-ED-Leon, leon*^*1*^, *leon*^*19-2*^ mutants were described previously [29, 58]; *UAS-Bsk-DN* and *UAS-Puc* were gifts from Jui-Chou Hsu (National Tsing Hua University, Taiwan). *UAS-Flag-Ubpy* was generated by subcloning *Ubpy* from SD04548 (*Drosophila* Genomics Resource Center (DGRC) into the pUAST vector. To monitor autophagy in *Drosophila* larval fat bodies, early third instar larvae fed on normal food or starved in 20% sucrose for 4 h were collected for experiments. The larval fat body cell clones were generated using the FLP-out technique as described previously [59]. In CQ treatment experiments, second instar larvae were transferred to normal food supplemented with 10 mg/ml chloroquine (Sigma) for 6 h.

### Cell culture and transfection

HEK293T, HeLa and MCF7 cells (obtained from ATCC) were cultured at 37 °C in Dulbecco’s Modified Eagle Medium (DMEM) with 10% fetal bovne serum (FBS) and 1% antibiotic (penicillin-streptomycin). PolyJet^TM^ DNA in vitro transfection reagent (Signagen) were used for transfection. The PolyJet reagent and DNA (1:2 ratio) were mixed and diluted in serum free DMEM for 10 ~ 15 min at room temperature. The PolyJet-DNA mixture was then added to the subconfluent cell culture for cell transfection.

### Plasmids and shRNAs

GST-tagged Leon-WT, Leon-ED, and Leon truncated mutants were generated by PCR and subcloned into pGEX-4T3. HA-USP5 was generated by subcloning USP5 into pcDNA3.1. Flag-tagged Atg1 and ULK1 were previously described [58]. The lentiviral shRNA clones used to knockdown USP5 were obtained from the National RNAi Core Facility of Academia Sinica. The target sequence for shUSP5#1 is 5ʹ-CTTTGCCTTCATTAGTCACAT-3ʹ (TRCN0000004066) and for shUSP5#2 is 5ʹ-GATAGACATGAACCAGCGGAT-3ʹ (TRCN0000293539). The scramble shRNA was used as the control.

### Antibodies

Antibodies used for this study were anti-LC3 (Cell Signaling, 4108), anti-LC3 (Novus, NB100-2220), anti-Ref(2)P (Abcam, ab178440), anti-GABARAP (Abcam, ab109364), anti-pULK1-Ser555 (Cell signaling, 5869), anti-ULK1 (Cell signaling, 8054), anti-USP5 (Proteintech, 10473-1-AP), anti-p62 (MBL, PM045), anti-Flag M2 (Sigma, F1804), anti-MYC (Santa Cruz, SC-40), anti-HA (Sigma, H9658), anti-ACTB/β-actin (Novus, NB600-501), anti-tubulin (Sigma, T0198), anti-GFP (Abcam, ab290), anti-ubiquitin (FK2; Sigma, ST1200), and anti-ubiquitin (P4D1; Cell signaling, 3936). To generate antiserum against *Drosophila* Atg1, the C-terminal segment of Atg1 (amino acid residues 562-855) was cloned to pET32a vector to generate 6xHis-tagged fusion protein. The fusion protein was purified using a Ni Sephsrose 6 FF column (GE Healthcare). The purified protein was used to immunize rabbits by LTK BioLaboratories and the resulting antiserum was purified by NAb Protein A Plus Spin Kit (Thermo).

### Immunofluorescence

Cells grown to subconfluence were fixed with cold methanol for 10 min, permeabilized with 0.1% Triton X-100 in PBS, blocked with 5% BSA, and incubated overnight at 4 °C with the primary antibodies in PBS with 5% BSA. Cells were then washed twice in PBS and incubated with secondary antibodies at room temperature for 1 h. Cells were mounted in Fluoromount-GTM with DAPI (Invitrogen) and images were acquired using Olympus FV3000 Confocal Microscopy with an UPlanXApo 60X/1.42NA objective. The *Drosophila* fat bodies were dissected from second instar larvae, fixed with 4% paraformaldehyde for 30 min, permeabilized with 0.3% Triton X-100 in PBS, and blocked with 5% normal goat serum in 0.1% Triton-PBS. Tissues were incubated overnight with the primary antibodies in PBS with 5% goat serum, and 0.3% Triton X-100. After washing twice in PBS and incubating with secondary antibodies, tissues were stained with DAPI and mounted in 90% glycerol in PBS. For LysoTracker staining, larvae fat body were dissected in PBS and immediately incubated in PBS with 1 μM LysoTracker Red DND-99 (Invitrogen) and DAPI. Samples were washed in PBS and mounted on slides. Images were acquired with a confocal laser scanning microscope (Olympus FV3000).

### Immunoprecipitation and immunoblotting

Cells transfected with the indicated plasmids were washed with PBS and scraped from dishes in lysis buffer (50 mM Tris–HCl, pH 7.4, 150 mMNaCl, 1 mM EDTA, 10% glycerol, 0.5% triton x-100, 10 mM NaF,1 mM phenylmethylsulfonyl fluoride (PMSF) and protease inhibitor cocktail (Roche, Indianapolis, IN, USA) and lysed for 20 min at 4 °C. Cell lysates were incubated with primary antibodies overnight at 4 °C and then with protein G-Sepharose beads (GEHealthcare) for 1 h at 4 °C. Beads were washed with lysis buffer and boiled with SDS sample buffer for 10 min. For protein extraction from *Drosophila* larval fat bodies, twenty second instar larvae were dissected in PBS, and fat bodies were lysed in SDS sample buffer. Samples were resolved by SDS-polyacrylamide gel electrophoresis (PAGE) and transferred to Immobilon-P polyvinylidene diflouride membrane (Millipore). Membranes were incubated with indicated primary antibodies overnight at 4 °C, washing, and then incubated with HRP-conjugated secondary antibodies for 1 h at room temperature. Immunoblots were detected with ECL reagent (Millipore) and quantified using ImageJ software (NIH).

### Statistical analysis

All data points were presented as mean ± SEM. Statistical analysis was performed by Student’s t test for comparisons between two groups. Comparisons between more than two groups were performed using ANOVA and Tukey’s multiple comparison tests in GraphPad Prism 7.0. Differences were considered significant if *p*-values were less than 0.05 (*), 0.01 (**), 0.001 (***), 0.0001 (****).

## Supplementary information

Supplementary information

checklist

## Data Availability

The data supporting the findings of this study are available within the paper and its supplementary information.
