# Supplementary material for: The deubiquitinase Leon/USP5 interacts with Atg1/ULK1 and antagonizes autophagy
Source: Cell Death Dis. 2023 Aug 22;14(8):540. doi: 10.1038/s41419-023-06062-x (PMC10444890; doi:10.1038/s41419-023-06062-x)

Supplementary information

Methods

Drosophila stocks, genetics and treatment

Flies were raised at 25°C following standard procedures. The *Drosophila* RNAi strains used in Table S1 were as follows: *CG32479* (BL36897), *CG4166* (BL28725) were obtained from Bloomington Stock Center. *CG8445* (v107757, v47743), *CG4265* (v103614, v26468), *CG3431* (v32443, v103481), *CG15817* (v41605, v100992), *CG14619* (v104382, v37929), *CG12082* (v17568), *CG1490* (v110324, v18231), *CG5798* (v107623), *CG1945* (v107716, v30679), *CG7023* (v100586, v27802), *CG5384* (v110227, v27405), *CG8494* (v42609), *CG3016* (v110616), *CG30421* (v33727), *CG8334* (v18982), *CG5794* (v106192, v27517), *CG5505* (v105989, v11152), *CG8830* (v28960), *CG7288* (v47664, v110535), *CG4165* (v110286, v41977), *CG5486* (v103743, v26027), *CG2904* (v106671), *CG5603* (v101414, v15340), *CG8209* (v100483, v35858), *CG3781* (v108379, v7113), *CG4968* (v21978), *CG3251* (v100532, v34574), *CG9448* (v24030), *CG12190* (v105283, v52138), *CG18174* (v19273), *CG2224* (v20852, v108622), *CG4751* (v45530) were obtained from VDRC stock center.

RT-PCR and RT-qPCR

Total RNA was extracted from control or USP5 knockdown MCF7 cell cells utilizing TRIzol (Invitrogen) and 1 μg RNA from individual samples were reverse transcribed into cDNA using iScript cDNA synthesis kit (Bio-Rad) following the manufacturer's instructions. For RT-PCR analysis, we used I μg cDNA as the template and added primers, 2XsuperRed PCR Master Mix. Primers were used foe RT-PCR are shown on the list. The PCR product were loaded into an agarose gel for electrophoresis. Real-time PCR was conducted in triplicates with SYBR green kit (Bio-Rad). The mRNA expression of the indicated gene was normalized to the levels of GAPDH transcript and fold changes were quantified using the comparative CT method.

RT-PCR Primer list

Primer (*Drosophila*)	sequences
Atg1_F	5'-CCGTGTCGCCAGTTGACAT-3'
Atg1_R	5'-GTTCTTTGGCACCAGCACAA-3'
Ref(2)P_F	5'-ATGCCGGAGAAGCTGTTGAA-3'
Ref(2)P_R	5'-ATCAGCGTCGATCCAGAAGG-3'

Act5C_F	5'-TTGTCTGGGCAAGAGGATCAG-3'
Act5C_R	5'-ACCACTCGCACTTGCACTTTC-3’

Primer (human)	sequences
ATG5_F	5'-AAGCAACTCTGGATGGGATT-3'
ATG5_R	5'-GCAGCCACAGGACGAAAC-3'
ULK1_F	5'-GGCAAGTTCGAGTTCTCCCG-3'
ULK1_R	5'-CGACCTCCAAATCGTGCTTCT-3'
ATG13_F	5'-GAAACAAGGGCATGA ATA-3'
ATG13_R	5'-CACTGTCCCAACACGAAC-3'
SQSTM1_F	5'-ATCGGAGGATCCGAGTGT-3'
SQSTM1_R	5'-TGGCTGTGAGCTGCTCTT-3'

RT-qPCR Primer list

Primer (human)	sequences
ATG5_F	5'-AGACCTTCTGCACTGTCCATC-3'
ATG5_R	5'-GCAATCCCATCCAGAGTTGCT-3'
ULK1_F	5'-TGCCCCTGGTTGAATGTTCT-3'
ULK1_R	5'-ACACCAGCCCAACAATTCCA-3'
ATG13_F	5'-CCGAAAAGTGGGGGCTTTTG-3'
ATG13_R	5'-TCGGTATCCTCCAGCTCCAA-3'
SQSTM1_F	5'-ATCGGAGGATCCGAGTGT-3'
SQSTM1_R	5'-TGGCTGTGAGCTGCTCTT-3'

Drosophila DUBs RNAi screen														
DUB (human ortholog)	Fed	Star.	DUB (human ortholog)	Fed	Star.	DUB (human ortholog)	Fed	Star.	DUB (human ortholog)	Fed	Star.	DUB (human ortholog)	Fed	Star.
CG8445 (BAP1)	N.S	N.S	CG5798/ UBPY (USP8)	+	N.S	CG3016 (USP30)	N.S	N.S	CG4165 (USP45)	+	N.S	CG3251 (OTUD4)	N.S	N.S
CG4265/Uch (UCHL3)	N.S	N.S	CG1945/faf (USP9X)	N.S	N.S	CG30421 (USP31/43)	N.S	N.S	CG5486/ Ubp64E (USP47)	N.S	N.S	CG9448/ Trbd (ZRANB1)	N.S	N.S
CG3431/Duch37 (Uch-L5)	N.S	N.S	CG32479 (USP10)	N.S	N.S	CG8334 (USP32)	N.S	N.S	CG2904/ec (USP54)	N.S	N.S	CG12190/ RYBP (YAF2)	N.S	N.S
CG15817 (USP1)	N.S	N.S	CG7023 (USP12)	N.S	N.S	CG5794 (USP34)	N.S	N.S	CG5603 (CYLD)	N.S	N.S	CG18174/ Rpn11 (PSMD14)	N.S	N.S
CG14619 (USP2/21)	N.S	N.S	CG5384 (USP14)	N.S	N.S	CG5505/ scrawny (USP36)	+	N.S	CG8209 (UBXN1)	N.S	N.S	CG2224 (AMSH)	N.S	N.S
CG12082/ Leon (USP5)	+	N.S	CG8494 (USP20/33)	N.S	N.S	CG8830 (USP35)	N.S	N.S	CG3781 (JOSD2)	N.S	N.S	CG4751 (MPND)	N.S	N.S
CG1490/ Dusp7 (USP7)	N.S	N.S	CG4166/not (USP22/51)	N.S	N.S	CG7288 (USP39)	-	-	CG4968 (OTUB1)	N.S	N.S			

Star.: starvation +: increased Atg8 puncta -: decreased Atg8 puncta N.S: No significant change
:UCH family :USP family :MJD family :OUT family :JAMM family

Table S1. RNAi-mediated knockdown of *Drosophila* DUBs involved in the regulation of autophagy in the larval fat body under fed and starvation conditions.

Fig S1

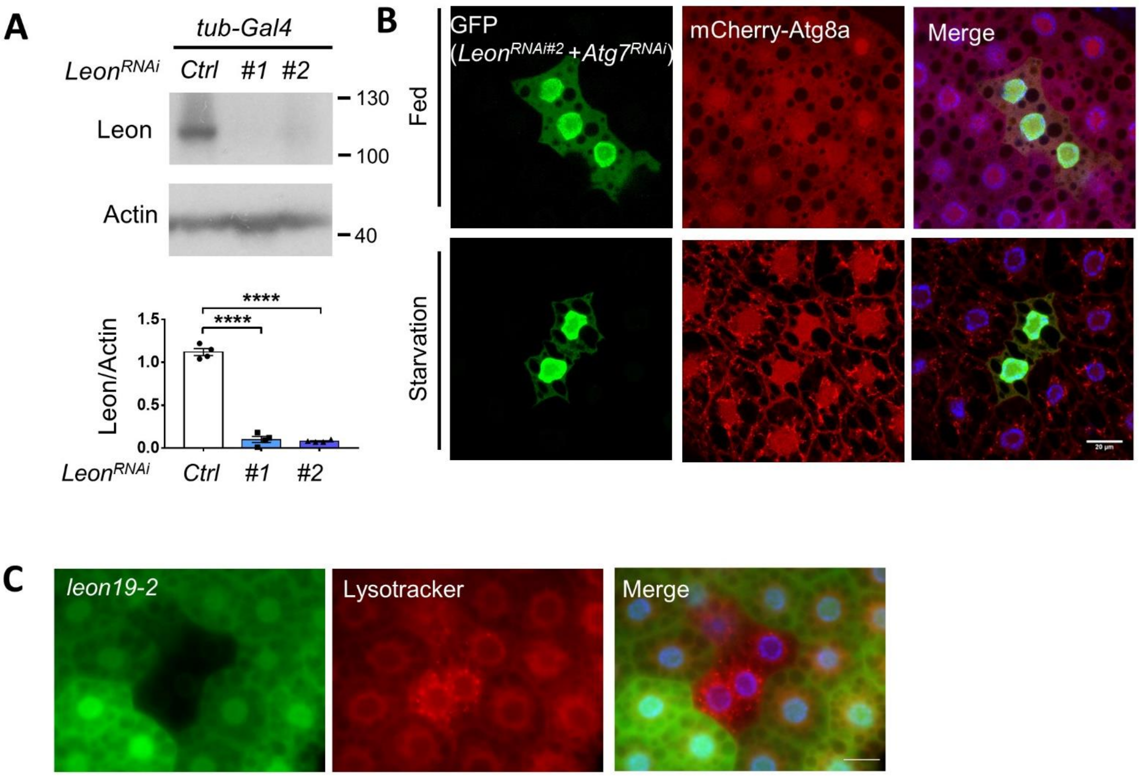

Fig S1. Knockdown of *Leon* promotes autophagy.

Western blot analysis of Leon expression levels in control and Leon depleted animals. Blots are representative of three independent experiments. Data are presented as means ± SEM. *P<0.05; ***P<0.001. (B) Clonal knockdown of *Atg7* in larval fat bodies blocked *Leon*^{RNAi}-induced Atg8a puncta formation under fed and starvation conditions. (C) Confocal microscopy analysis of *leon*$^{19\text{-}2}$ homozygous mutant fat body cell clones (GFP^{-}) stained with LysoTracker. GFP expression marks wild-type clones. Scale bar, 20μm.

Fig S2

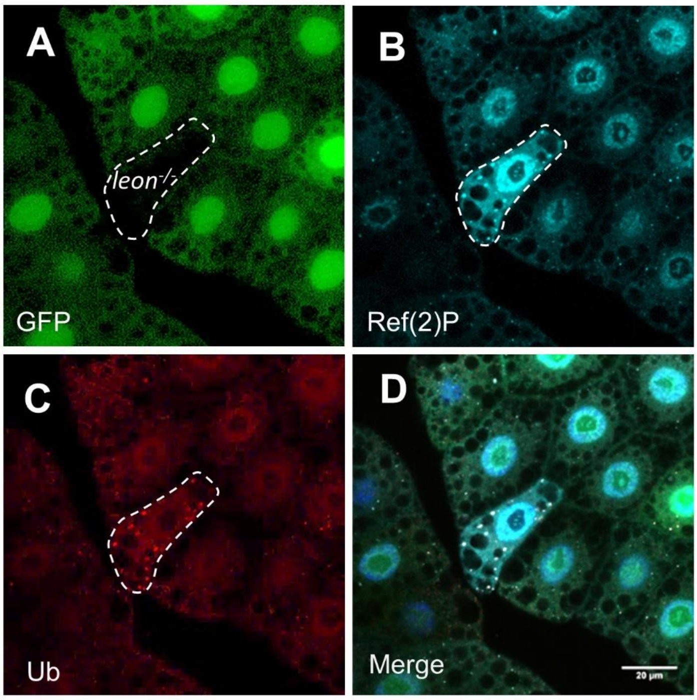

Fig S2. Loss of *leon* leads to accumulation of Ref(2)P puncta and ubiquitinated protein aggregates.

(A-D) Confocal microscopy analysis of *leon*$^{19-2}$ homozygous mutant fat body cell clones (GFP^{-}) stained with Ref(2)P (B) and ubiquitin (C) antibodies. Dashed line indicates the *leon*$^{19-2}$ homozygous mutant cell. GFP expression marks wild-type clones. Scale bar, 20μm.

Fig S3

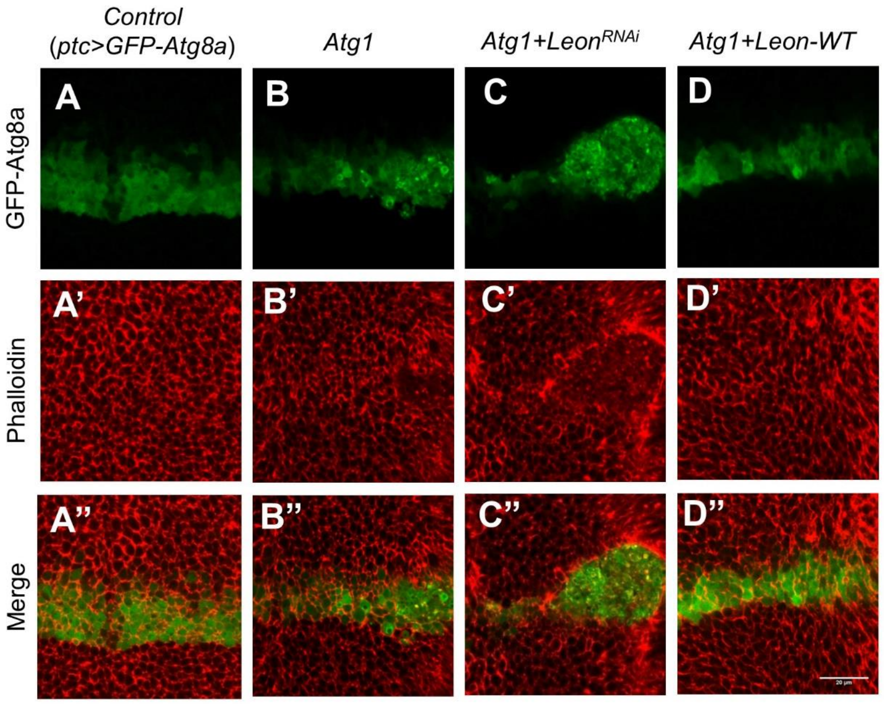

Fig S3. Overexpression of *Leon* suppresses *Atg1*-induced autophagy and actin cytoskeletal defects.

(A-D”) Third-instar wing imaginal discs from *ptc-Gal4>UAS-GFP-Atg8a* controls or flies expressing indicated transgenes were stained with phalloidin. (A-D) Co-expression of *Leon-WT* but not *Leon*^{RNAi} suppressed *Atg1*-induced Atg8a puncta formation. (A’-D’) Co-expression of *Leon-WT* but not *Leon*^{RNAi} suppressed *Atg1*-induced actin cytoskeletal defects. Scale bar, 20μm.

Fig S4

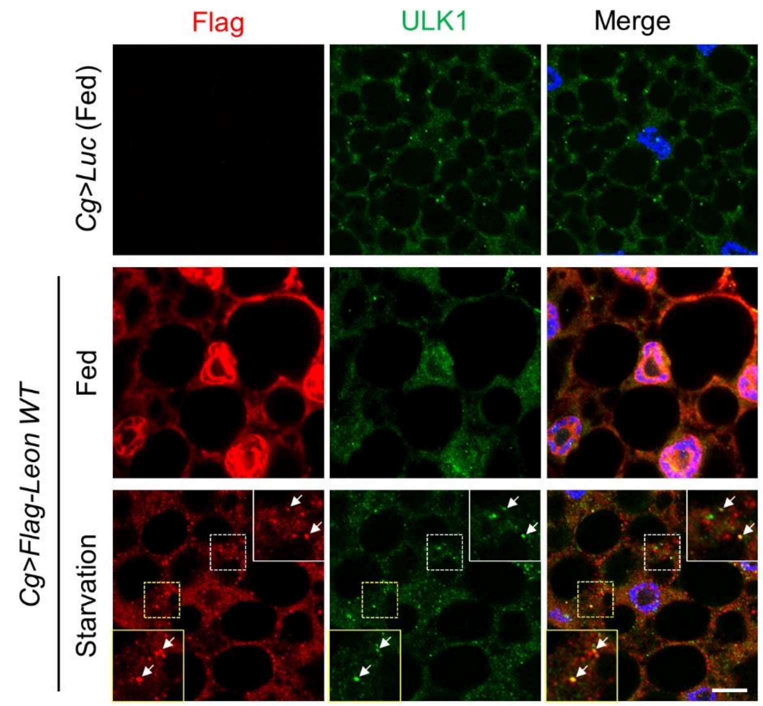

Fig S4. Leon is partially co-localized with Atg1/ULK1 in cytoplasm during nutrient starvation.

Immunostaining of fed or starved *Drosophila* early third instar larval fat bodies expressing control (*Luc*) or *Flag-Leon* by *Cg-Gal4* with anti-Flag and anti-ULK1 [1] antibodies. Co-localization of Leon and Atg1/ULK1 are highlighted by arrows (yellow and white insets). Scale bar, 10μm.

Fig S5

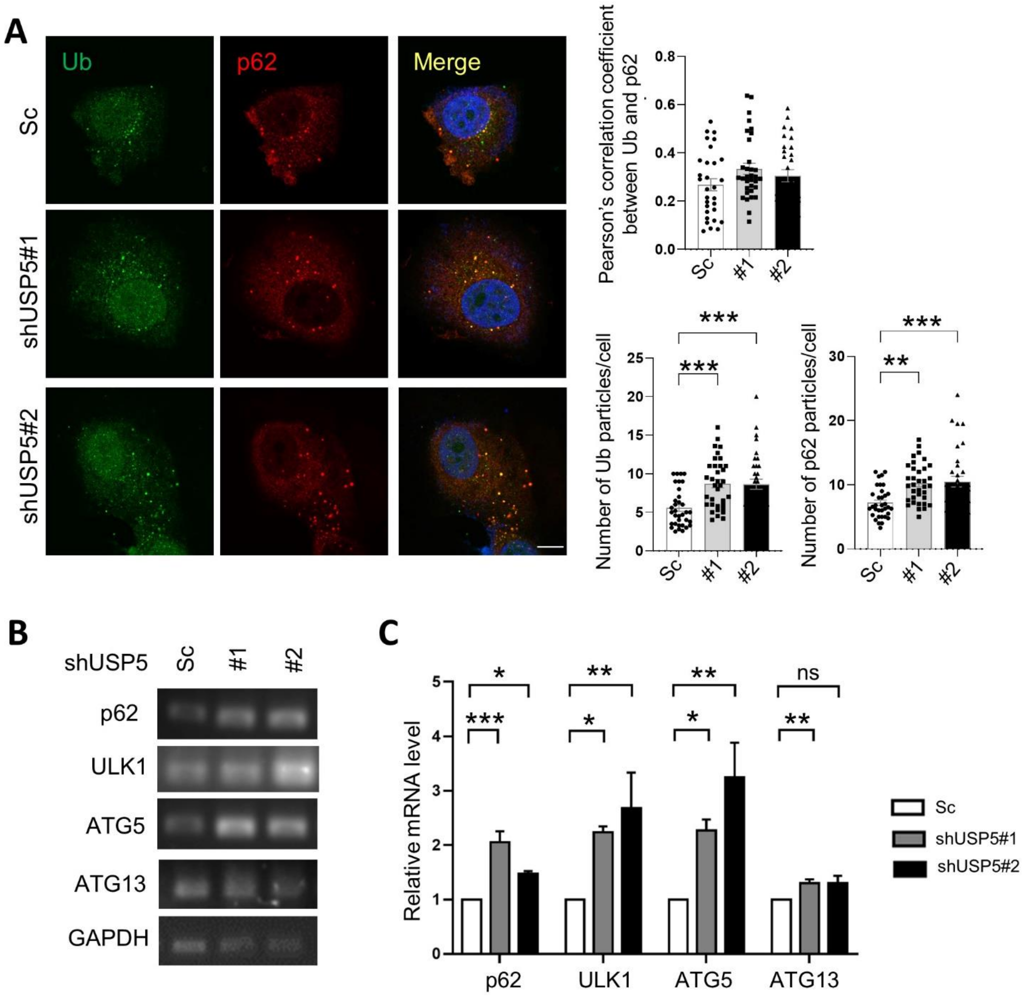

Fig S5. USP5 knockdown results in increased levels of p62 puncta and ubiquitinated protein aggregates.

(A) Immunofluorescence analysis of p62 and ubiquitinated protein aggregates in control or USP5 knockdown MCF7 cell. Quantification of p62 and polyubiquitin puncta in cells. Data are presented as means ± SEM, n≥ 30 cells of three independent experiments, Scale bar, 10μm; The Pearson՝s correlation coefficient was analyzed by ImegeJ. (B) RT-PCR analysis of *p62*, *ULK1*, *ATG5*, *ATG13* and *GAPDH* gene expression in control or USP5 knockdown MCF7 cells. (C) RT-qPCR analysis of *p62*, *ULK1*, *ATG5*, *ATG13* gene expression in control or USP5 knockdown MCF7 cells. The data are normalized to the level of *GAPDH* gene expression. Data are presented as means ± SEM of 4 independent experiments. *P < 0.05; **P < 0.01; *** P < 0.001.

Fig S6

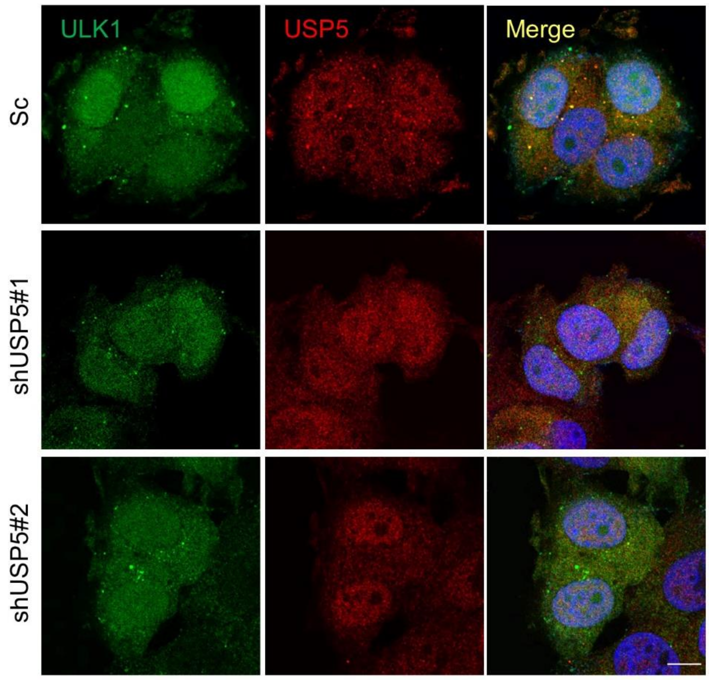

Fig S6. Mammalian USP5 is partially co-localized with ULK1 in cytoplasm. Immunofluorescence analysis of ULK1 and USP5 localization in control or USP5 knockdown MCF7 cells. ULK1 and USP5 were detected with indicated antibodies. Scale bar, 10μm.

Reference

1. Zhang S, Yi S, Wang L, Li S, Wang H, Song L, Ou J, Zhang M, Wang R, Wang M, Zheng Y, Yang K, Liu T, Ho MS. Cyclin-G-associated kinase GAK/dAux regulates autophagy initiation via ULK1/Atg1 in glia. Proc Natl Acad Sci USA. 2023;120(29):e2301002120.

Fig1

E

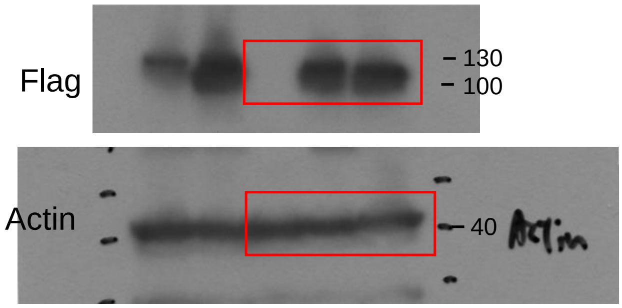

Fig2

C

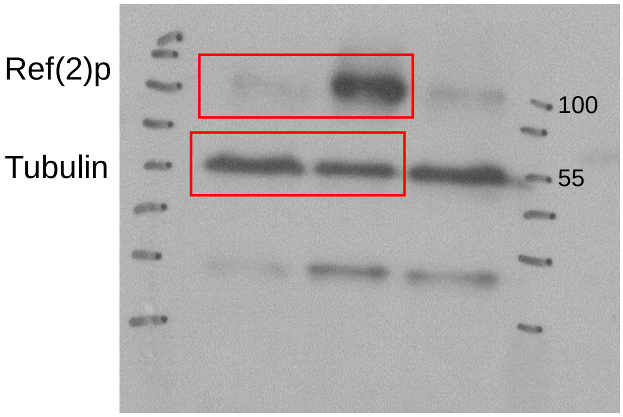

D

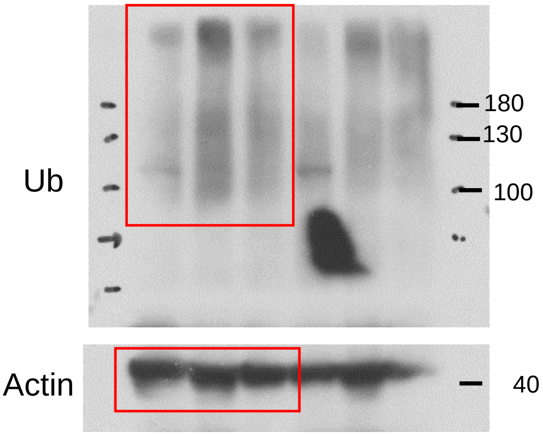

Fig5

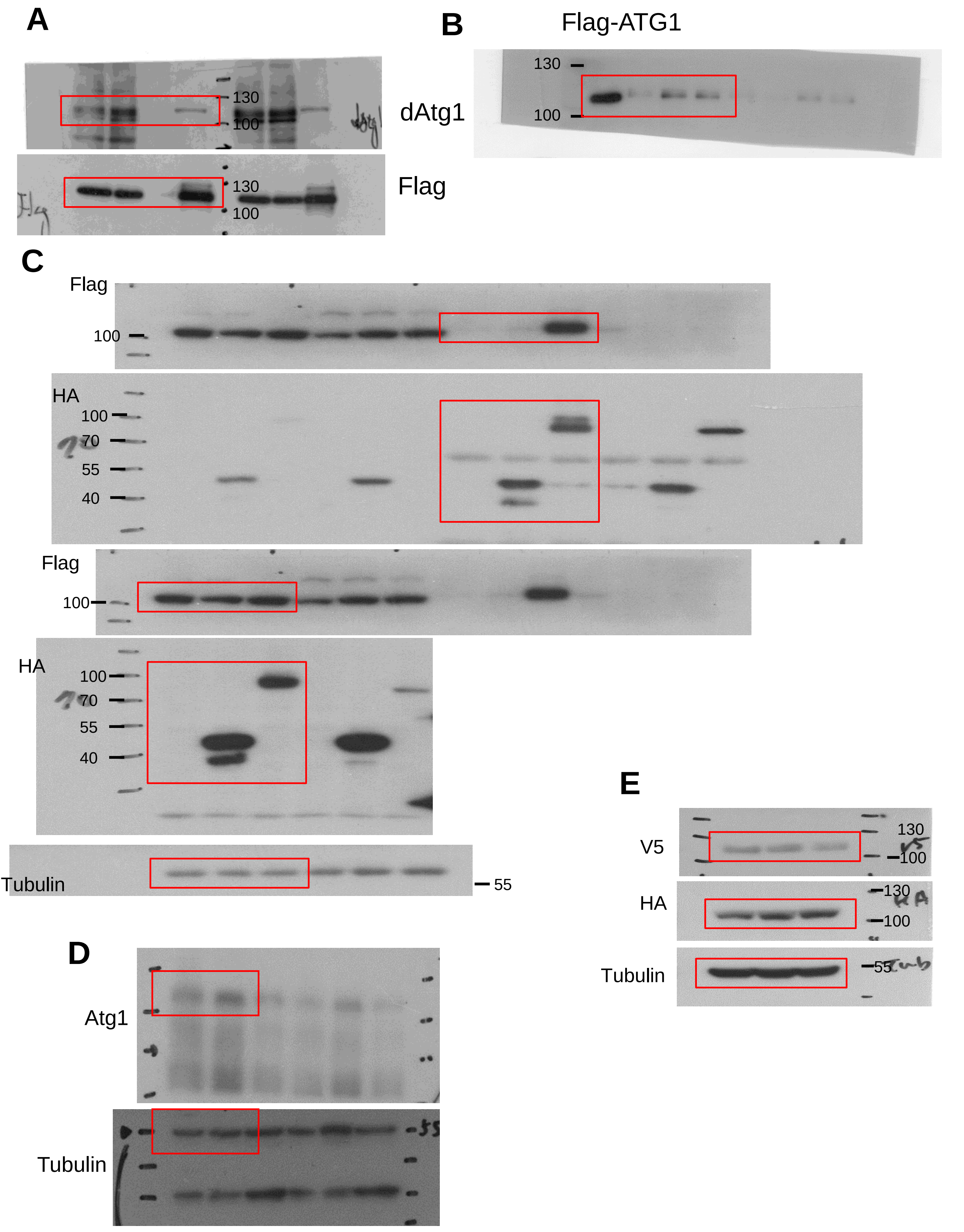
A
130
100
dAtg1
Flag
130
100
B
Flag-ATG1
130
100
C
Flag
100
HA
100
70
55
40
Flag
100
HA
100
70
55
40
Tubulin
55
D
Atg1
Tubulin
E
V5
130
100
HA
130
100
Tubulin
55

Fig6

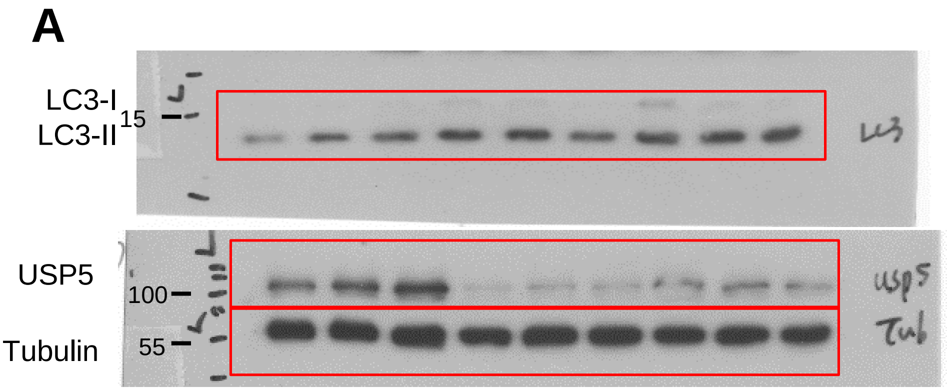

Fig7

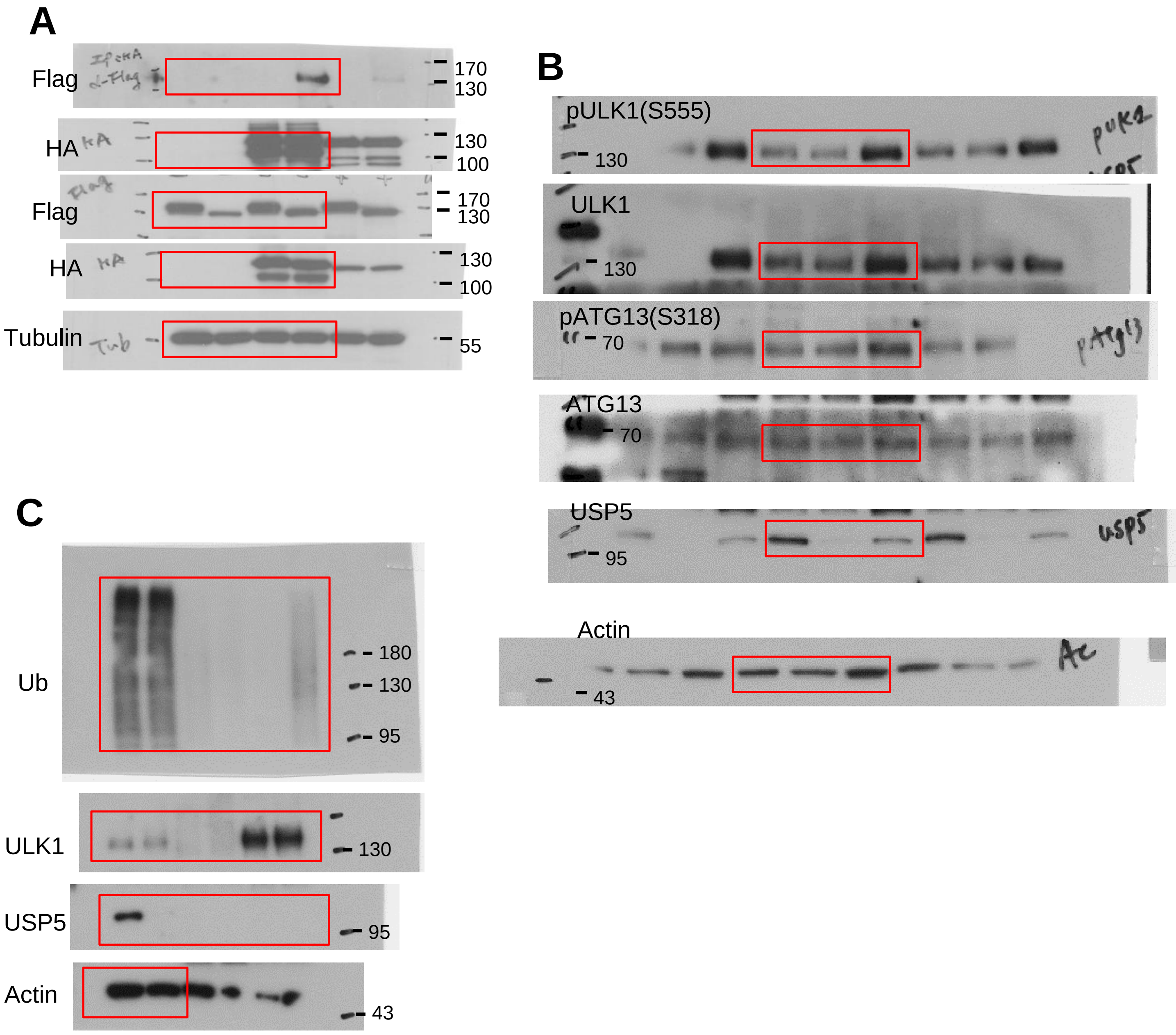
A
Flag
170
130
HA
130
100
Flag
170
130
HA
130
100
Tubulin
55
B
pULK1(S555)
130
ULK1
130
pATG13(S318)
70
ATG13
70
USP5
95
Actin
43
C
Ub
180
130
95
ULK1
130
USP5
95
Actin
43

FigS1

A

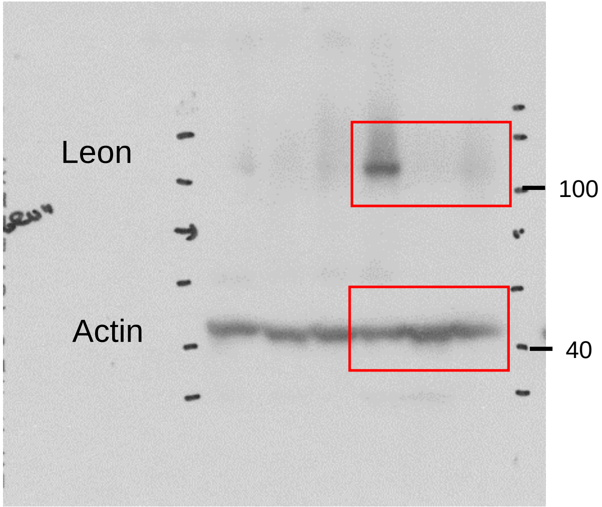

Supplement: Supplementary file 1 — Supplementary information [file 41419_2023_6062_MOESM1_ESM.pdf]
